# Silicon mitigates heavy metal stress by regulating P-type heavy metal ATPases, *Oryza sativa* low silicon genes, and endogenous phytohormones

**DOI:** 10.1186/1471-2229-14-13

**Published:** 2014-01-09

**Authors:** Yoon-Ha Kim, Abdul Latif Khan, Duk-Hwan Kim, Seung-Yeol Lee, Kyung-Min Kim, Muhammad Waqas, Hee-Young Jung, Jae-Ho Shin, Jong-Guk Kim, In-Jung Lee

**Affiliations:** 1 School of Applied Biosciences, College of Agriculture and Life Science, Kyungpook National University, Daegu 702-701, Republic of Korea; 2 Department of Biological Science & Chemistry, University of Nizwa, Nizwa 616, Oman; 3 Department of Life Sciences and Biotechnology, Kyungpook National University, Daegu 702-701, Republic of Korea

**Keywords:** Silicon, Heavy metal stress, Root physiology, Phytohormones, P-type heavy metal ATPase, *Oryza sativa*, Low silicon

## Abstract

**Background:**

Silicon (Si) application has been known to enhance the tolerance of plants against abiotic stresses. However, the protective mechanism of Si under heavy metals contamination is poorly understood. The aim of this study was to assess the role of Si in counteracting toxicity due to cadmium (Cd) and copper (Cu) in rice plants (*Oryza sativa*).

**Results:**

Si significantly improved the growth and biomass of rice plants and reduced the toxic effects of Cd/Cu after different stress periods. Si treatment ameliorated root function and structure compared with non-treated rice plants, which suffered severe root damage. In the presence of Si, the Cd/Cu concentration was significantly lower in rice plants, and there was also a reduction in lipid peroxidation and fatty acid desaturation in plant tissues. The reduced uptake of metals in the roots modulated the signaling of phytohormones involved in responses to stress and host defense, such as abscisic acid, jasmonic acid, and salicylic acid. Furthermore, the low concentration of metals significantly down regulated the mRNA expression of enzymes encoding heavy metal transporters (*OsHMA2* and *OsHMA3*) in Si-metal-treated rice plants. Genes responsible for Si transport (*OsLSi1* and *OsLSi2*), showed a significant up-regulation of mRNA expression with Si treatment in rice plants.

**Conclusion:**

The present study supports the active role of Si in the regulation of stresses from heavy metal exposure through changes in root morphology.

## Background

Exposure to heavy metal toxicity has become a major limiting factor in the growth and yield of crop plants, affecting the sustainability of agricultural production and hence threatening food security. Heavy metal toxicity retards plant growth by marginalizing the cellular functions of proteins, lipids, and elemental components of thylakoid membranes. Disturbances in the thylakoid membranes, organelles indispensable for photosynthetic activity, are often correlated with senescence processes [1-3]. Among heavy metals, cadmium (Cd) and copper (Cu) have been known to hinder the growth of crop plants, especially rice plants. Rice paddies are contaminated by Cd/Cu in phosphate fertilizers, sludge, and irrigated water. Cd absorbed and transported inside rice plants can create severe health problems because rice is consumed on a daily basis [4-7]. Cu bioaccumulation inside plant tissues tends to disturb the enzymatic activities required for chlorophyll biosynthesis [2]. In addition, Cd/Cu influences leaf elongation, cell wall elasticity, potassium levels, and sugar accumulation [8].

Cadmium (Cd) and copper (Cu) are taken together with other elements (such as K, Ca, Mg, and Fe), through the transmembrane carrier from the root cortex to the stele [9,10]. The uptake of heavy metals is influenced by metal-transporting transmembrane proteins, including Heavy Metal ATPases (HMAs), Low-affinity Cation Transporters (LCTs) and Iron-regulated Transporters (IRTs) [7,11,12]. These commonly known metal transport genes are located throughout the structure of plant which translocate a diverse set of metal ions [13,14]. Previous studies in *Arabidopsis thaliana* have confirmed the physiological functions of the HMAs (AtHMA1, AtHMA2, AtHMA3, and AtHMA4) that is to detoxify zinc from chloroplast and signal Cd accumulation in the vacuoles and plasma membrane [15-18].

Cd/Cu causes the formation of reactive oxygen species (ROS) [10] that damage membrane permeability and function. Peroxides of polyunsaturated fatty acids generate malondialdehyde (MDA), the most abundant aldehydic lipid breakdown product [19] that indicates the levels of stress and injury to the functional membrane. To counteract these of stress conditions, plant hormones also play a central role. For example, jasmonic acid (JA) and salicylic acid (SA) are involved in defense-related signaling during stress conditions. In addition, abscisic acid (ABA), a stress-responsive hormone, is involved with stomatal closure to ensure plants do not lose a substantial amount of water [20].

To mitigate and reduce the negative effects of heavy metals, various prospects have been evaluated. Silicon (Si) has been found to serve as a beneficial element for plant growth and development [21]. It is the second most abundant element in soil and readily absorbed; terrestrial plants contain it at an appreciable concentration, 1% to 10% or even higher in plant dry matter [21]. Si is an essential component of rice plants and its accumulation is helpful in maintaining sustainable production [22]. The physiological functions of Si have been studied extensively and Si is known as an essential constituent of plants and fertilizers [21,23-26]. Numerous studies have revealed that Si is a beneficial element to higher plants, particularly grasses and various cultivated crops like rice, wheat, tomato, and cucumber [21,23,27-30]. Over the last decade, studies have revealed the ability of Si to mitigate various biotic (plant diseases and pests) and abiotic stresses (heavy metals, drought, and salinity) in crop plants [20]. Ma et al. [31] has reported that Si influx and efflux strongly regulate a set genes, *Oryza Satica Low Silicon Rice 1* and *2* (*OsLsi1* and *OsLsi2*), in the plasma membrane of rice plant cells. *OsLsi1* is localized at the distal side of the cell while *OsLsi2* is confined to the proximal side of the same cell and both are arranged on casparian strips [31]. The role of these two genes in Si transport and stress conditions is still unknown. To understand these interactions, the aim of this study was to assess the role of *OsLsi1* and *OsLsi2* during heavy metal (Cd and Cu) exposure to rice plants under Si treatments. We also investigated the role of *HMAs* during heavy metal toxicity and their regulation during the application of Si. In response to metal stress, the regulation of the phytohormones salicylic acid, abscisic acid, and jasmonic acid were also analyzed.

## Results

### Si improves rice plant growth under heavy metal exposure

**Figure 1:**
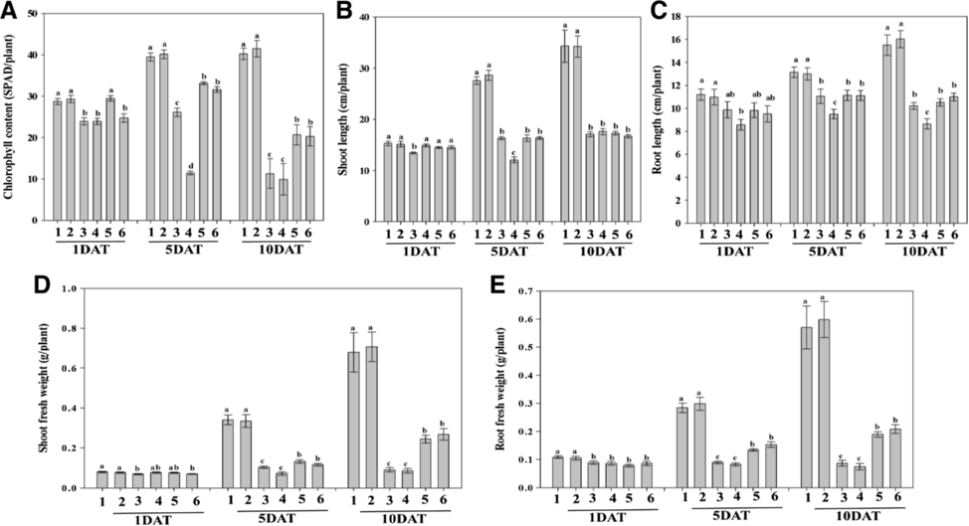
**Effects of heavy metal (Cu/Cd) with and without Si (1.0 mM) application on physiological parameters of rice plants.** Surveys of all the physiological parameters were recorded after 1, 5 and 10 days treatments (DAT). In the figure, number indicated different treatments (1- Control; 2-Si; 3- Cu; 4- Cd; 5- Cu + Si; 6- Cd + Si). **A** - chlorophyll contents; **B** - Shoot length; **C** - Root length; **D** - Shoot fresh weight; **E** - Root fresh weight of the rice plants. Bars represent means of 81 plants that is the sum of three replications per treatment ± standard error. Means denoted by the same letter were not significantly different at *P > 0.05* by Duncan Multiple Range Test (DMRT).

The effect of Si and heavy metal (Cu and Cd) treatments on rice plant growth was assessed after different time points (1, 5, and 10 days after treatment – DAT; Figure 1). The results showed that Si application significantly increased plant growth attributes as compared with the control plants (non-Si) under normal growth conditions. After 24 h exposure to heavy metal stress, Cu significantly affected the shoot and root lengths of rice plants as compared to Cd. After 5 and 10 days of Cu or Cd treatments, the shoot growth was significantly reduced as compared with control and Si treatment (Figure 1).

Metal stress has significantly decreased the root length of rice plants as compared to control plants under normal growth conditions. Treatment with Cd, in particular, significantly affected the root architecture and reduced the root length as compared with plants treated with Cu, Cu + Si or Cd + Si after 1, 5, and 10 DAT. In case of chlorophyll content, after one day, Si and Si + Cu had no significant difference as compared to control, but these treatments had higher chlorophyll than Cd, Cu, and Cd + Si treatments. After 5 and 10 days of treatment, the chlorophyll content was significantly higher in Si-treated plants as compared to metal stressed plants (Figure 1).

The heavy metals treatment has adversely affected the biomass (shoots and roots) and phenotypic characters of rice plants. Although, there was no significant effect on the biomass or phenotypic characteristics after Si and non-Si treated metal stress after day 1 (Figure 1). However, we observed a significant difference after 5 and 10 days of metal stress in Si-treated plants, where a significantly higher biomass (in fresh weight) of shoots and roots was observed. The biomass of the shoots and roots was also significantly different with Si treatment as compared to non-metal-treated control plants. In terms of phenotypic characteristics, leaf damage and necrosis were pronounced with Cd treatment (compared with Cu treatment) after 5 and 10 DAT. Phenotypic characteristics were also significantly ameliorated with Si application after 5 and 10-DAT because Si treatment with metal stress showed relatively lower rates of necrosis and leaf damage.

### Si affects heavy metal transport in the roots of rice plants

Cd/Cu ions concentration was measured in the rice roots after the application of metal stress and Si. Cd concentration was undetected in the control, Si and Cu treatments after 1, 5, or 10 days. However, Cd accumulation in rice plants with Cd-treatment was significantly higher than Si + Cd at 1, 5, and 10-DAT (Table 1). The Cd concentration was approximately 2.3, 2.4 and 1.9 fold higher in the Cd-treated rice compared to Si + Cd at 1, 5, and 10-DAT, respectively.

**Table 1:** Ion concentrations of Cd, Cu and Si in the rice root under stress

**Treatments**	**Ion concentrations (μmol g**^**-1**^**)**		
	**Cd**	**Cu**	**Si**
**1-DAT**			
Control	ND	20.8 ± 2.1 c	201.32 ± 3.21 d
Si	ND	37.8 ± 4.6 c	765.8 ± 39.8 c
Cu	ND	2777.4 ± 207.1 a	677.6 ± 22.7 c
Cd	611.5 ± 78.0 a	39.2 ± 2.1 c	788.1 ± 58.4 c
Cu + Si	ND	1374.0 ± 179.6 b	3482.6 ± 155.9 a
Cd + Si	263.2 ± 14.2 b	28.0 ± 3.6 c	2104.2 ± 17.3 b
**5-DAT**			
Control	ND	24.5 ± 1.2 d	234.2 ± 5.2 d
Si	ND	45.0 ± 4.9 c	600.5 ± 8.9 c
Cu	ND	2398.3 ± 88.0 a	612.8 ± 65.7 c
Cd	934.0 ± 49.4 a	45.6 ± 4.7 c	797.7 ± 54.2 c
Cu + Si	ND	1903.4 ± 14.8 b	1778.8 ± 35.4 a
Cd + Si	399.6 ± 15.7 b	37.7 ± 4.1 c	1383.7 ± 49.8 b
**10-DAT**			
Control	ND	31.3 ± 6.1 d	119.2 ± 2.4 d
Si	ND	48.8 ± 6.9 c	126.1 ± 9.0 d
Cu	ND	1433.8 ± 105.2 a	167.1 ± 14.3 c
Cd	680.7 ± 30.9 a	51.0 ± 2.3 c	170.6 ± 45.0 c
Cu + Si	ND	977.8 ± 16.8 b	390.9 ± 19.4 a
Cd + Si	342.0 ± 42.9 b	49.5 ± 0.6 c	231.1 ± 61.7 b

Mean ± standard error from three replications per treatment. *ND*, Not detected; *DAT*, Days after treatment. In the column, the same letters indicate not significant difference (*P > 0.05*) by Duncan’s Multiple Range Test (DMRT).

The Cu concentration was not significantly different in the control, Cd, and Si + Cd treated plants at 1, 5, and 10-DAT. On the other hand, Cu accumulation was significantly higher in the Cu-treated plants as compared with the Si + Cu plants after 1, 5, and 10-DAT. The Cu accumulation was approximately 2.0, 1.2 and 1.7 fold higher in Cu-treated plants compared with the Si + Cu at 1, 5, and 10-DAT, respectively.

Si accumulation in rice roots was significantly different in Cd and Cu-treated rice plants compared to the control after 1, 5 and 10-DAT. However, the level of Si accumulation was significantly higher in plants treated with Si + Cu, or Si + Cd after 1, 5, and 10 DAT. Overall, the Si + Cu treatment accumulated Si in higher quantities in the roots compared to the Si + Cd rice plants after 1, 5, and 10-DAT (Table 1).

### Si improves root morphology during metal stress treatment

**Figure 2:**
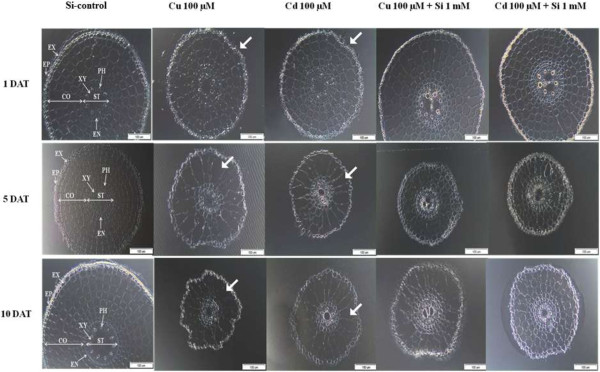
**Cross section of rice root under Cu/Cd and with and without Si application for 1, 5 and 10 days (DAT).** Abbreviation in figure showed like CO: cortex, EX: exodermis, EP: epidermis, EN: endodermis, PH: phloem, ST: stele, XY: xylem. Bars = 100 μm in all the figures.

To assess the effects of metal stress and Si treatment on root morphology and structure, sections (2 cm) from the rice roots were obtained from each sample at 1, 5, and 10-DAT. Light microscopic analysis showed that the exodermis (EX), epidermis (EP), endodermis (EN) and cortex regions of the plants treated with Cd or Cu were slightly affected as compared to Si-treated control roots at 1-DAT (Figure 2). The cellular spaces were wider in the non-Si-treated plant roots and the suberin lamellae were partially broken at instant places (arrow). In contrast, the suberin lamellae of the EX, EP, and cortex regions in the Si and metal-treated plants (Cu + Si and Cd + Si) were fully developed and lacked any visible deformities. In the cortex regions, the damage was more vigorous in Cu and Cu + Si compared with Cd and Cd + Si at 1-DAT (Figure 2).

The deleterious effects of Cd and Cu were more severe after 5 and 10-DAT. Although the stele and epidermal suberin lamellae were still intact, the EN and cortex regions were severely damaged and poorly differentiated. The root cellular apparatus was severely broken and damaged at 10-DAT after exposure to Cd and Cu stress. The Cu-treated roots, compared to Cd, were more deformed in shape and size; moreover, the Cu-treated tissue parts were poorly differentiated. In case of Si and metal treatment, endodermal suberin lamellae were closer to each other, while fewer walls were damaged in the Cd + Si plants compared to plants treated with Cd and Cu alone (Figure 2). In Cu + Si and Cd + Si treatments, the negative effects of metal stress were substantially less, although the cortex cellular lamellae were broken at various places. These effects were more pronounced in Cu + Si compared with Cd + Si treatments. After 10-DAT, the adverse effects were further evident in the Cu + Si plants than in the Cd + Si plants. Altogether, this indicates that Si application has an ameliorative role in the Cd stress regulation (compared to Cu) in root morphology of rice plants.

### Si regulates phytohormones during heavy metal stress

**Figure 3:**
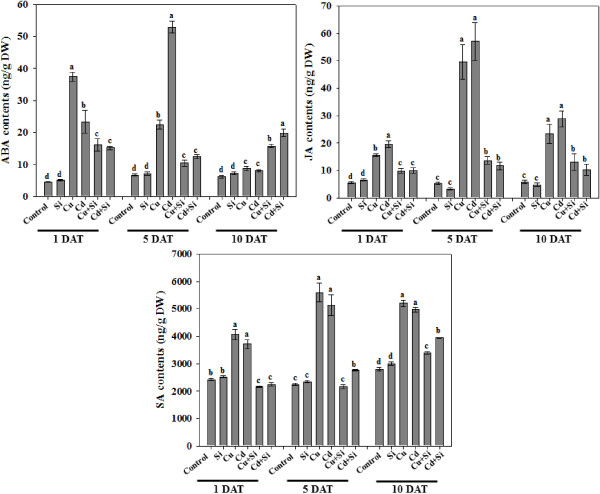
**Regulation of endogenous phytohormones in response to Si and heavy metal stress treatments.** The aerial parts of the rice plants were analyzed for signaling of abscisic acid (ABA), jasmonic acid (JA) and salicylic acid (SA) against 1, 5 and 10 days after treatments (DAT) of Si and metal. The hydroponic growth medium served as control. Bars represent means (of three replications) ± standard error. Means denoted by the same letter are not significantly different (*P > 0.05*) as evaluated by Duncan Multiple Range Test (DMRT).

Plant hormones were regulated during the stress conditions with or without the application of silicon. Upon Cd/Cu treatments, the endogenous abscisic acid (ABA) content was significantly increased in 1 and 5-DAT. ABA was initially higher with Cu treatments as compared to Cd; however, the opposite was observed at 5-DAT. Although the ABA levels were still higher compared with the Si-treated control plants at 5-DAT, the application of Si significantly lowered the ABA levels under metal stress. At 10-DAT, the Cu or Cd treatment had slightly higher ABA levels compared to the control (Figure 3). In Si treatment, the ABA content was significantly up-regulated with Si + Cd/Cu treatments compared to the control at 1 and 5-DAT and also with the sole Cd/Cu treatment at 10-DAT. The results suggest that Si initially counteracted the responses to heavy metal stress; however, with increasing stress periods, the stress-responsive ABA was exponentially activated.

The response pattern of endogenous jasmonic acid (JA) was different from ABA. In a previous study [30], Si application to rice plants reduced JA biosynthesis under wounding stresses. In this study, heavy metal stress caused a similar JA response. The JA content was significantly higher with sole Cd stress compared with Cu stress. In Si treatment, comparing the Si and Si + Cd/Cu treatment, the JA content from Cd/Cu treatments was significantly up-regulated. The JA level was not significantly different between the Si + Cd and Si + Cu treatments and the different stress periods did not affect the JA content during Si application. Our results suggest Si application reduces JA biosynthesis under heavy metal stress (Figure 3).

Silicon application increased the synthesis of salicylic acid (SA) depending on the duration of the Cd/Cu stress (Figure 3). The effect of SA biosynthesis was not significantly different between the Cd and Cu treatment alone at 1, 5, and 10-DAT. The SA content was 2 ~ 3 fold higher with Cu or Cd treatments than in the control, Si, Si + Cu, or Si + Cd treatments (Figure 3). Si application and metal stress had no significant effect on the SA quantities at 1-DAT, but at 5-DAT and 10-DAT, the SA content was significantly higher in Cd compared to Cu (Figure 3).

The results of our phytohormonal analyses showed that the content of ABA, SA and JA exponentially increase with Cd/Cu stress compared to Si treatments. Similarly, periodic exposure initially increased phytohormonal responses with Cd/Cu treatment compared to the Si treatments. We also observed cross-talk in the hormonal responses to Cd/Cu.

### Si decreases lipid peroxidation activity and affects fatty acid composition under heavy metal stress

**Figure 4:**
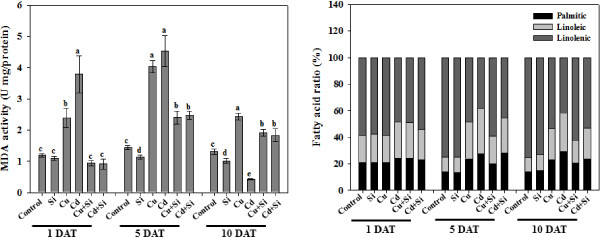
**Effects of heavy metal (Cu/Cd) with and without Si application on the lipid peroxidation (MDA) and fatty acid ratio.** The bars in MDA shows the mean ± standard error while the means denoted by the same letter are not significantly different (*P > 0.05*) as evaluated by Duncan Multiple Range Test (DMRT). The bars in fatty acid ratio represent the mean values of three replicates.

The level of lipid peroxidation was measured from the malondialdehyde (MDA) content (Figure 4). Lipid peroxidation was significantly higher with the Cd/Cu treatments at 1 and 5-DAT compared to Si + Cd/Cu. The MDA level in plants with Si and metal stress after 1-DAT was not significantly different from the control; however, at 5 and 10-DAT, the MDA levels were significantly higher compared to control. The analyses also showed low level of lipid membrane degradation with Si application compared to non-Si plants under metal stress (Figure 4).

To further analyze the lipid membrane damage, three fatty acids (palmitic acid (C 16:0), linoleic acid (C 18:2) and linolenic acid (C 18:3) were quantified in rice plants treated with metals and Si (Figure 4). The percentages of fatty acids at 1-DAT were not significantly different for all the treatments. At 5 and 10-DAT, however, the percentage of C 18:3 was significantly reduced with the Cd/Cu treatment compared to the control, and Cd/Cu with Si treatments had higher levels of C 18:3 compared to the Cd/Cu treatments alone (Figure 4). C 16:0 and C 18:2 showed the opposite trend; at 1-DAT, there were no significant differences between the treatments, but at 5 and 10-DAT, these fatty acids were significantly higher in the Cd/Cu-treatment compared to the Si + Cd/Cu-treatments (Figure 4).

### Si inhibits expressions of *OsHMA2* and *OsHMA3* under heavy metal stress

**Figure 5:**
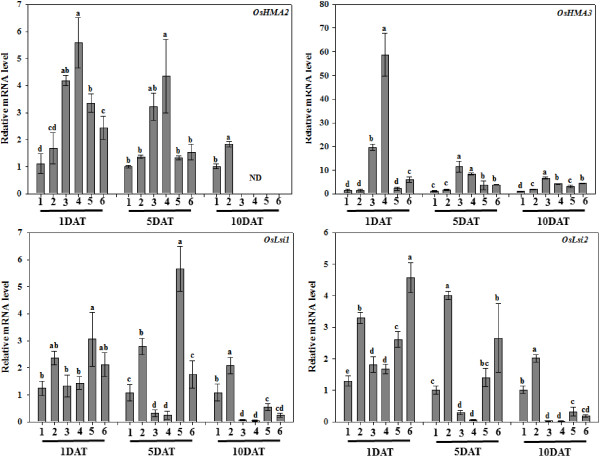
**The mRNA expression of heavy metal transporter (*OsHMA2 *and *OsHMA3*) and silicon accumulator (*OsLsi1 *and *OsLsi2*) enzymes in the root samples of Si and Cu/Cd treated rice plants.** In the figure, number at the *x-axis* indicate 1 – Control, 2 – Si, 3 – Cu, 4 – Cd, 5 – Si + Cu, 6 – Si + Cd. Bars represent means (of three replications) ± standard error. Means denoted by the same letter were not significantly different at *P > 0.05* by Duncan Multiple Range Test (DMRT).

According to previous reports of Hussain et al. [32], Andrés-Colás et al. [33], Courbot et al. [16], Lee et al. [14], Nocito et al. [34], and Satoh-Nagasawa et al. [12], heavy metal ATPases (encoded by the *HMA* genes) are key regulators of the heavy metal stress responses and transport in most of the higher plants. We assessed the expressions of *HMA* genes (*OsHMA2* and *OsHMA3*) in rice plants. The mRNA expressions of *OsHMA2* and *OsHMA3* were analyzed in Si, non-Si and Cd/Cu treatments (Figure 5). The relative expression of *OsHMA2* at 1-DAT was significantly higher with Cd/Cu treatments compared to Si-treatments. *OsHMA2* was also higher with the Cd/Cu + Si treatments compared with sole Si and control but was significantly lower than the Cd/Cu treatments without Si. A similar tendency was observed at 5-DAT; however, at 10-DAT, *OsHMA2* was not exponentially expressed compared with the other treatments. The mRNA expression of *OsHMA3* was similar at 1 and 5-DAT, but after 10-DAT, it was significantly increased in the non-Si Cu rice plants compared with Si + Cd/Cu and Cd-treated plants (Figure 5).

### Si increases the expression of *OsLsi1 and OsLsi2* under heavy metal stress

The *Oryza sativa* low silicon genes (*OsLsi1* and *OsLsi2*) regulate Si influx and efflux in the roots of rice plants [31,35]. To assess the heavy metal stress-induced regulation of these two genes with Si, we determined the mRNA expression level of *OsLsi1* and *OsLsi2* in rice plants (Figure 5). The expression level of *OsLsi1* at 1-DAT revealed no significant differences between the Si alone, control, and Cd/Cu treatments; however, it was exponentially expressed in the Si + Cd/Cu-treated plants (Figure 5). Similarly, the relative mRNA levels of *OsLsi1* were significantly down-regulated with Cd/Cu application at 5-DAT but highly up-regulated with Si and Si + Cd/Cu application (Figure 5). At 10-DAT, the expression level of *OsLsi1* was down-regulated with the Cd/Cu treatments compared to the Si-treated plants. In the Si + Cd/Cu treatments, *OsLsi1* was also more expressed than with Cd/Cu treatments alone (Figure 5).

A similar expression pattern was observed for *OsLSi2*. The relative mRNA level of *OsLsi2* at 1-DAT was up-regulated with Cd/Cu alone and Si + Cd/Cu treatment than in the Si-treated control. However, the expression of *OsLsi2* was more highly expressed with Si + Cd/Cu treatment compared to the Cd/Cu plants without Si (Figure 5). At 5-DAT, the relative mRNA level of *OsLsi2* was significantly down-regulated with the Cd/Cu treatments compared with the control, Si alone and Si + Cd/Cu-treated plants (Figure 5). At 10-DAT, the relative mRNA level of *OsLsi2* was significantly down regulated in Cd/Cu and Cd/Cu + Si than in the control and Si alone. Si application during Cd/Cu stress significantly activated the *OsLsi2* enzymes to counteract the negative effects of the heavy metals. Because there was no stress during the treatment with Si alone, *OsLsi1* and *OsLsi2* maintained their expression at 5 and 10-DAT.

## Discussion and conclusions

Heavy metals released into the environment tend to accumulate in soils and become available to plants through their roots [36,37]. In rice paddy fields, the heavy metals Cd and Cu are readily absorbed into rice roots, which are transported to the shoot parts through the symplastic pathway [4-7,38-40]. An increased concentration of Cd/Cu causes the inhibition of cellular processes such as photosynthesis, electron transport, and lipid peroxidation due to the binding of these metals to sulfhydryl groups [2,41,42].

We found that Cd/Cu application significantly affects the growth of rice plants. The rice plants had a reduction in chlorophyll content after exposure to Cd/Cu. Chlorophyll degradation further reduced the shoot length and biomass of the rice plants after different heavy metal stress periods. In addition, the rice plants had a lower root length and biomass after an exposure to Cd/Cu stress. Langer et al. [43] and Liu et al. [44] previously found that heavy metals applied to rice plants caused a very weak root growth pattern and morphology while Si ameliorated this impact. In *Brassica napus*, Cd at a low concentration (5 μM) reduced the plant growth, chlorophyll content, and photosynthesis, leading to stomatal closure [45]. We observed similar negative effects in the root structure of the Cd/Cu-treated rice plants. However, these adverse effects were greatly minimized with exogenous Si-application. Various physiological parameters, such as shoot and root length, biomass, and chlorophyll content were significantly higher in the Si-treated plants compared to the control plants under heavy metal stress.

Our results further suggest that Si-treatment reduces the accumulation of heavy metals in rice roots. The accumulation of Cd/Cu inside the root tissues of Cd/Cu-treated plants was significantly higher compared with the Si + Cd/Cu-treatments. The effects of Si-application on the alleviation of Cd toxicity in our experiment were comparable to previous reports where 1.8 mM of exogenous Si increased the fresh weight of rice seedlings grown in solutions contaminated with Cd [46]. The non-Si-treated plants, on the other hand, were significantly affected by the negative effects of metal stress, as revealed in our micrographs of the rice roots. The root cell structure of the epidermis, exodermis, and cortex were seriously damaged with increasing durations of Cu or Cd stress, while the root structure was less affected by Si treatment. In addition, Cd/Cu accumulation was much more pronounced with Cd/Cu treatment alone compared to the Si-treated plants. This likely reflects the binding of heavy metals in a stable complex with further distribution inside the root, an activity minimized with the Si treatments compared to the non-Si plants. The possible mechanisms for Si inhibition of metal transport in plants may be due to the thickening of the casparian strips in the endodermis and cell wall of the xylem causing the deposition of lignin and Si in the cell walls of the dermal regions [47,48].

In addition, excessive Cu induces leaf chlorosis due to peroxidative breakdown of the pigments and lipid membrane [5]. We found that Si modulated both leaf chlorosis and lipid peroxidation in rice plants. Lipid peroxidation indicates oxidative stress damage on the membrane due to metal toxicity [49-51]. The results demonstrate that lipid peroxidation was significantly higher in the Cd/Cu rice plants compared to the Si-control; however, MDA generation with Si + Cd/Cu treatments was significantly reduced. The degree of lipid peroxidation is usually measured by the concentration of secondary breakdown products derived from these initial hydroperoxides. Peroxides of polyunsaturated fatty acids generate malondialdehyde (MDA) on decomposition, and MDA is typically the most abundant individual aldehydic lipid breakdown product [52]. It has been estimated that more than 75% of the measured MDA is derived from α-linoleic acid [53]. Metal stress significantly reduced the percentage of α-linoleic acid, suggesting a higher attack from reactive oxygen species (ROS) at the lipid membrane compared to plants provided with Si. We conclude that having high concentrations of α-linolenic acid in chloroplasts and mitochondria, via the production of small oxygenated compounds such as MDA, signal and absorb ROS generated in these organelles. This proposed route for the elimination of ROS would be hemi-metabolic because it is initiated by non-enzymatic fatty acid fragmentation [54].

Alpha-linolenic acid, on the other hand, serves as a precursor of JA, a potent lipid molecule essential for signaling defenses during stress [30]. JA can act synergistically or antagonistically with other hormones, like salicylic acid (SA). SA plays a key role in regulating physiological processes, such as plant resistance to biotic and abiotic stresses [55-57], protection from ROS through antioxidant production, induction of gene expression, and absorption and distribution of elements under heavy metal stress conditions [57-59]. Higher levels of SA suggest an increased mitigation of ROS during metal stress. Interestingly, we found that Si treatment significantly lowers SA modulation during metal stress. While there was also a significant reduction of JA in the Si-treated plants under metal-induced stress, this is opposite to the effects we observed for Si on lipid peroxidation and fatty acid saturation. The release of α-linolenic acid from plant membrane lipids by stress-activated lipases is thought to provide a substrate for lipoxygenase and the subsequent synthesis of JA [54]. Because the Si-treated plants were less affected by metal stress, perhaps the rice plant is able to consume less α-linolenic acid for synthesizing JA. However, these dynamics are still unclear and extensive studies on Si and JA modulation during stress periods are required.

Abscisic acid (ABA), on the other hand, was significantly lower in Si-treated plants at 1-DAT and 5-DAT, but then increased at 10-DAT in the Si + metal-treated plants compared to the metal-treated plants. ABA plays an important role during many phases of the plant life cycle, including seed development and dormancy, and plant responses to various environmental stresses [20,60]. Some studies have reported that ABA content increases in plants exposed to Cu and Cd pollution [61], resulting in reduced stomatal conductance and hence, affecting photosynthesis and chlorophyll synthesis. These effects were ameliorated with Si treatment during metal stress and thus less ABA was formed. In our results, the ABA level was significantly reduced with Si treatment. ABA has an antagonistic behavior with JA/SA biosynthesis during Si treatment, suggesting an active role of ABA during Si and stress application. This is further confirmed by gene expression profile indicating salinity stress and ABA biosynthesis (unpublished data).

Our results also showed that *OsLsi* expressions were significantly higher in Si-treated plants at 1, 5, and 10-DAT during Cd/Cu stress, while in the non-Si-treated plant these were not expressed or expressed at low levels. The results suggest an active accumulation of Si via the roots, permitting its efficient deposition in the roots and shoots (data not shown). *OsLsi2* is mainly expressed in the root parts at the plasma membrane [31]. Si deposition in the roots provides additional strength to the root structure for counteracting the intruding toxic metal ions, revealed in our micrographs after Cd/Cu stress. Large amounts of Si aggregate in the exodermis and endodermis [62] and after 1 and 5-DAT, we observed less damage to the exodermis and endodermis regions of the roots compared to the non-Si plants during Cd/Cu stress. Our results suggest a higher accumulation of Si in the roots reduced the influx of heavy metal. Thus, heavy metal transport was reduced in the Si treated plants compared to the non-Si plants. Similar results were revealed in the expression analysis of *OsHMA2* and *OsHMA3*. The results also showed that during Cu/Cd stress, the *OsLsi* genes were not activated vigorously compared to *OsHMA3*. However, after Si application in combination with the Cu/Cd treatments, both *OsHMA3* and *OsLsi* genes activated significantly to counteract the negative impacts of the metal stress. This further evidence supports our results of increase Si accumulation and amelioration of the plant growth attributes. This synergistic activation of *OsHMA3* and *OsLsi* genes also showed that during metal stress, the root morphology was less disturbed compared to sole application of Cu/Cd. In conclusion, the findings of our study suggest a protective mechanism of Si during heavy metal stress. These results suggest that the application of Si may be an effective method for controlling Cd/Cu transfer from contaminated paddy soil into the food chain.

## Methods

### Plant material, Si and heavy metal application

Rice Seeds (*Oryza sativa* L. cv Dongjinbeyo) were procured from the National Institute of Crop Science, Rural Development Administration, Republic of Korea. Seeds were surface sterilized with 5% sodium hypochlorite for 15 min and thoroughly washed with autoclaved double distilled water. The rice seeds were grown on autoclaved sand medium for seven days to obtain equal length seedlings. Rice seedlings were then transplanted to hydroponic media and further grown till twenty-one days in growth chamber. The growth chamber (KGC-175 VH, KOENCON) conditions were programmed for a 14-hour light (08:00 ~ 22:00; 30°C; relative humidity 70%) and 10-hour dark (22:00 ~ 08:00, 25°C; relative humidity 70%) cycle. Yoshida solution [63] was used as a growth medium for the rice seedlings in plastic pots (25 × 20 × 20 cm). Treatments were arranged factorially in a randomized experimental design with 27 plants per treatment. The treatments included (i) control (growth medium), (ii) sole Si, (iii) cadmium (Cd)/copper (Cu) and (iv) Si with Cd/Cu.

The twenty-one-day-old rice plants were treated with Si (Na_2_SiO_3_; 1.0 mM). The pH (5.1 ± 0.1) of the rice growth medium was maintained by adding HCl to inhibit the polymerization of the silicates [64]. An equivalent amount of Na (as NaCl) was also added to the zero Si-treated plants to compensate for the Na content of 1.0 mM in the Si-treated plants. Heavy metal stress was applied to the root zone of the rice plants by adding 100 μM cadmium (CdCl_2_)/copper (CuSO_4_). The metal stress was applied periodically for 1, 5, or 10 days. Each treatment was repeated in triplicate.

Rice plants were harvested after stress treatments, immediately frozen in liquid nitrogen, and shifted to -80°C. Prior to biochemical and hormonal analysis, plant samples were freeze-dried using a Virtis Freeze Dryer (Gardiner, NY, USA) for 4–7 days while for other experiments (such as mRNA expression analysis) fresh plant samples were used. The fresh shoot and root length were recorded after the treatment period. The chlorophyll contents were measured through a chlorophyll meter (SPAD-502 Minolta, Japan).

### Determination of the Cd, Cu, and Si concentration in rice roots

After 1, 5, and 10 days, the Cd and Cu-treated rice plant roots (with or without Si application) were thoroughly washed with double distilled water. The roots were then soaked in 0.5 M HCl for 20 s, rinsed with double distilled water [65], and dried for 72 hours in an oven at 80°C. Rice root samples were weighted and ground to a fine powder, and then digested in 5 ml of a tertiary mixture of HNO_3_ : H_2_SO_4_ : HClO_4_ (10: 1: 4 (v/v/v)). The contents of Cd and Cu in the rice roots were determined with inductively coupled plasma (ICP) (Optima 7900DV, Perkin-Elmer, USA).

### Microscopic analysis

Rice seedlings’ roots were fixed at 4°C in Karnovsky’s fixative (2% glutaraldehyde, 1% paraformaldehyde, in 0.05 M sodium cacodylate, pH 7.2). Samples were thoroughly washed two times in 0.05 M sodium cacodylate buffer. The specimens were then dehydrated through a gradient ethanol series (30-50-70-80-90%-absolute ethanol, 15 min each), and cleared in propylene oxide. Finally, the specimens were embedded in Spurr’s epoxy resin. The resin was polymerized in a dry oven at 70°C for 8 hours. Semi-thin sections were obtained using an ultra-microtome (MT-7000, RMC, USA) and then the sections were observed by differential interference contrast microscopy (Olympus, Japan).

### Abscisic acid extraction and quantification

The endogenous ABA content was quantified from the frozen samples by following the protocols of Qi et al. [66] and Kamboj et al. [67]. Aerial parts of the plant samples were extracted with 30 ml of extraction solution containing 95% isopropanol, 5% glacial acetic acid, and 20 ng of [(±)–3,5,5,7,7,7–d6]–ABA. The filtrate was concentrated by a rotary evaporator. The residue was dissolved in 4 ml of 1 N sodium hydroxide solution, and then washed three times with 3 ml of methylene chloride to remove lipophilic materials. The aqueous phase, brought to approximately a pH of 3.5 with 6 N hydrochloric acid was partitioned three times into ethyl acetate (EtOAc). EtOAc extracts were then combined and evaporated. The dried residue was dissolved in phosphate buffer (pH 8.0) and then run through a polyvinylpolypyrrolidone (PVPP) column. The phosphate buffer was adjusted to pH 3.5 with 6 N HCl and partitioned three times into EtOAc. EtOAc extracts were combined again and evaporated. The residue was dissolved in dichloromethane (CH_2_Cl_2_), and passed through a silica cartridge (Sep-Pak; Water Associates, Milford, Massachusetts, USA) pre-washed with 10 ml of diethyl ether: methanol (3:2, v/v) and 10 ml of dichloromethane. ABA was recovered from the cartridge by elution with 10 ml of diethyl ether (CH_3_-CH_2_)_2_O: methanol (MeOH) (3:2, v/v). The extracts were dried and methylated by adding diazomethane for GC/MS-SIM (6890 N network GC system, and the 5973 network mass-selective detector; Agilent Technologies, Palo Alto, CA, USA) analysis. For quantification, the Lab-Base (ThermoQuset, Manchester, UK) data system software was used to monitor responses to ions with an m/e of 162 and 190 for Me-ABA and 166 and 194 for Me-[^2^H_6_]-ABA.

### Jasmonic acid extraction and quantification

The endogenous JA level was quantified according to the protocol of McCloud and Baldwin [68]. The lyophilized aerial tissues parts were ground to a fine powder with a mortar and pestle. The powder (0.1 g) was suspended in a solution of acetone and 50 mM citric acid (70:30, v/v) and [9,10-^2^H_2_]-9,10-dihydro-JA (20 ng) was added as an internal standard. The extracts were allowed to evaporate overnight at room temperature to avoid the loss of volatile fatty acids. The resulting aqueous solutions were then filtered and extracted three times, each time with 10 mL of diethyl ether. The pooled extracts were then loaded on a solid phase extraction cartridge (500 mg of sorbent, aminopropyl). After loading, the cartridges were washed with 7.0 mL of trichloromethane and 2-propanol (2:1, v/v). The bound JA and the pertinent standard were eluted with 10 mL of diethyl ether and acetic acid (98:2, v/v). After evaporation of the solvents and esterification of the residue with excess diazomethane, the sample was adjusted to 50 μL with dichloromethane. The extracts were then analyzed by GC-MS (6890 N network GC system and the 5973 network mass selective detector; Agilent Technologies, Palo Alto, CA, USA). To enhance the sensitivity of the method, spectra were recorded in the selected-ion mode. For JA determination, the fragment ion was monitored at m/z = 83 amu corresponding to the base peaks of JA and [9, 10-^2^H_2_]-9, 10-dihydro-JA. The amounts of endogenous JA were calculated from the peak areas of JA compared to the corresponding standards. The experiment was repeated three times.

### Free salicylic acid extraction and quantification

Free SA was extracted and quantified as described by Enyedi et al. [69] and Seskar et al. [70]. Freeze-dried aerial tissue samples were ground to powder form, and 0.1 g was sequentially extracted with 90% and 100% methanol by centrifuging at 10,000 × g. The combined methanol extracts were vacuum-dried. The dry pellets were resuspended in 2.5 ml of 5% trichloroacetic acid, and the supernatant was partitioned with ethyl acetate/cyclopentane/isopropanol (49.5:49.5:1, v/v). The top organic layer, containing free SA, was transferred to a 4 ml vial and dried with nitrogen gas. The dry SA was again suspended in 1 mL of 70% methanol. High-performance liquid chromatography (HPLC) analyses were carried out on a Shimadzu fluorescence detector (Shimadzu RF-10AXL, excitation and emission detected at 305 and 365 nm, respectively) fitted with a C18 reverse-phase HPLC column (HP hypersil ODS; particle size, 5 μm; pore size, 120-Å water; Additional file 1: Table S1). The flow rate was 1.0 ml/min. The SA analyses were repeated three times.

### Determination of lipid peroxidation activity

The extent of lipid peroxidation was determined by the method of Ohkawa et al. [71]. For this assay, 0.2 ml of 8.1% sodium dodecyl sulphate, 1.5 ml of 20% acetic acid (pH 3.5), and 1.5 ml of 0.81% thiobarbituric acid aqueous solution were added in succession in a reaction tube. Then 0.2 ml of root tissue homogenate extracted was added to 10 mM phosphate buffer (pH 7.0). The mixture was then heated in boiling water for 60 min. After cooling to room temperature, a 5 ml butanol/pyridine (15:1 v/v) solution was added. The upper organic layer was separated, and the optical density of the resulting pink color was recorded at 532 nm using a spectrophotometer. Tetramethoxypropane was used as an external standard. The level of lipid peroxides was expressed as micromoles of malondialdehyde (MDA) formed/gram tissue weight. The experiments were repeated three times.

### Fatty acid analysis

The root tissues of rice plants (1 g) were treated with 10 ml of hexane. The samples were then placed in a shaking incubator (150 rpm) at 50°C for 2 days. The supernatant was separated by centrifugation (1,200 × g at 25°C) followed by transfer into new tubes. Hexane was evaporated by passing air in an evaporating unit. The extracted material from each sample was placed in a screw-capped vial, and 5 ml of methylation solution (H_2_SO_4_/methanol/toluene 01:20:10 ml) were added. The sealed vial was heated in a water bath (100°C) for 60 min and allowed to cool to room temperature. Then 5 ml of water was added and shaken. The mixture was separated into two layers, and the upper layer was taken by Pasteur pipette and dried using anhydrous sodium sulphate for 5 min. One microliter of sample was directly injected into the GC using an automatic sampler (Agilent 7683B). GC-MS analysis was carried out on the Agilent Model 7890A series (Agilent, Dover, DE, USA) equipped with an Agilent 5975C MS detector and an Agilent 7683 autosampler, and a MS ChemStation Agilent v. A.03.00 was used. The GC-MS was equipped with a DB-5MS capillary column (30 m × 0.25 mm i.d. × 0.25 μm film thickness; J&W Scientific-Agilent, Folsom, CA, USA), while helium was used as a carrier gas with a flow rate of 0.6 ml/min in split mode (1:50). The injector temperature and detector temperature were 120 and 200°C, respectively. The column temperature was programmed from 50 to 200°C at 10°C/min and then finally held at 200°C for 5 min. The mass conditions were as follows: ionization voltage, 70 eV; scan rate, 1.6 scan/s; mass range, 30–450; and ion source temperature, 180°C. The components were identified based on their relative retention time and mass spectra compared with standards, Wiley7N, NIST library data of the GC-MS system, and data from the literature. The values of the fatty acids (palmitic acid, stearic acid, oleic acid, linoleic acid, and linolenic acid) were calculated in milligram/gram × 10^-1^.

### RNA extraction and RT-PCR analysis

The root samples of rice plants treated with heavy metals and Si were harvested in liquid nitrogen (after 1, 5, or 10 days) and immediately shifted to -80°C. Total RNA was extracted from rice plant samples using an RNeasy plant extraction minikit (Qiagen) according to the manufacturer’s instructions. First-strand cDNA was synthesized from 1 μg of total RNA using an oligo (dT) 18 primer and the SuperScript first strand synthesis system was used for the reverse transcriptase polymerase chain reaction (RT-PCR). RT-PCR was performed in a 50 μl solution containing a 1 μl aliquot of the cDNA reaction, 0.2 μM of gene-specific primers, 10 mM dNTPs, and 1 unit of rTaq DNA polymerase. PCR conditions included 30 cycles of denaturation at 95°C for 45 s, annealing at 53°C for 45 s and extension at 72°C for 90 s. PCR products were barely visible when the agarose gels were stained with ethidium bromide. These products were separated by electrophoresis on a 1.0% agarose gel, blotted onto a nylon membrane, and hybridized with a ^32^P-labeled probe specific to each gene that was generated from the non-conserved 3′ end regions. The RT-PCR analyses were performed according to the method of Lee et al. [14], Ma et al. [31,35] and Nocito et al. [34] (Additional file 2: Table S2). *OsAct1* was used as a standard gene.

### Statistical analysis

The data was statistically analyzed for standard deviation and error using Sigma Plot software (2004). The mean values were compared using Duncan’s multiple range tests at *P* < 0.05 (ANOVA SAS release 9.1; SAS, Cary, NC, USA).

## Abbreviations

Cd: Cadmium; Cu: Copper; HMAS: Heavy metal ATPase; LCTs: Low-affinity cation transporter; IRTs: Iron regulated transporter; ROS: Reactive oxygen species; MDA: Malondialdehyde; JA: Jasmonic acid; SA: Salicylic acid; ABA: Abscisic acid; Si: Silicon; OsLsi: *Oryza sativa* low silicon; EX: Exodermis; EP: Epidermis; EN: Endodermis; DAT: Day after treatment; ICP: Inductively coupled plasma.

## Competing interests

The authors declare that they have no competing interests.

## Authors’ contributions

YHK and ALK designed and performed the experiments. DHK, SYL and HYJ performed the microscopy analysis for the work. KMK, MW, JGK and JHS performed the molecular analysis. YHK, ALK and IJL wrote the manuscript. All authors read and approved the final manuscript.

## Supplementary Material

Additional file 1: Table S1 HPLC conditions used for salicylic acid analysis. Click here for file

Additional file 2: Table S2 List of primers used for RT-PCR analysis. Click here for file

## References

[B1] Lin CC Chen LM Liu ZH Rapid effect of copper on lignin biosynthesis in soybean roots Plant Sci 2005 168 855 861 10.1016/j.plantsci.2004.10.023

[B2] Maksymiec W Signalling responses in plants to heavy metal stress Acta Physiol Plant 2007 29 177 187 10.1007/s11738-007-0036-3

[B3] Molas J Changes of chloroplast ultrastructure and total chlorophyll concentration in cabbage leaves caused by excess of organic Ni(II) complexes Environ Exp Bot 2002 47 115 126 10.1016/S0098-8472(01)00116-2

[B4] Shah K Kumar RG Verma S Dubey RS Effect of cadmium on lipid peroxidation, superoxide anion generation and activities of antioxidant enzymes in growing rice seedlings Plant Sci 2001 161 1135 1144 10.1016/S0168-9452(01)00517-9

[B5] Liu JG Liang JS Li KQ Zhang ZJ Yu BY Lu XL Yang JC Zhu QS Correlations between cadmium and mineral nutrients in absorption and accumulation in various genotypes of rice under cadmium stress Chemosphere 2003 52 1467 1473 10.1016/S0045-6535(03)00484-3 12867177

[B6] Kikuchi T Okazaki M Toyota K Motobayashi T Kato M The input–output balance of cadmium in a paddy field of Tokyo Chemosphere 2007 67 920 927 10.1016/j.chemosphere.2006.11.018 17207840

[B7] Takahashi R Ishimaru Y Senoura T Shimo H Ishikawa S Arao T Nakanishi H Nishizawa NK The OsNRAMP1 iron transporter is involved in Cd accumulation in rice J Exp Bot 2011 62 4843 4850 10.1093/jxb/err136 21697258 PMC3192999

[B8] Patra M Bhowmik N Bandopadhyay B Sharma A Comparison of mercury, lead and arsenic with respect to genotoxic effects on plant systems and the development of genetic tolerance Environ Exp Bot 2004 52 199 223 10.1016/j.envexpbot.2004.02.009

[B9] Tudoreanu L Phillips CJC Modeling cadmium uptake and accumulation in plants Adv Agron 2004 84 121 157

[B10] Pál M Horváth E Janda T Páldi E Gabriella S Physiological changes and defense mechanisms induced by cadmium stress in maize J Plant Nutr Soil Sci 2007 169 239 246

[B11] Uraguchi S Kamiya T Sakamoto T Kasai K Sato Y Nagamura Y Yoshida A Kyozuka J Ishikawa S Fujiwara T Low-affinity cation transporter (OsLCT1) regulates cadmium transport into rice grains Proc Natl Acad Sci USA 2011 108 20959 20964 10.1073/pnas.1116531109 22160725 PMC3248505

[B12] Satoh-Nagasawa N Mori M Nakazawa N Kawamoto T Nagato Y Sakurai K Takahashi H Watanabe A Akagi H Mutations in rice (*Oryza sativa*) heavy metal ATPase2 (*OsHMA2*) restrict the translocation of zinc and cadmium Plant Cell Physiol 2012 53 213 224 10.1093/pcp/pcr166 22123790

[B13] Kuhlbrandt W Biology, structure and mechanism of P-type ATPases Nat Rev Mol Cell Biol 2004 5 282 295 10.1038/nrm1354 15071553

[B14] Lee SC Kim YY Lee YS An GH Rice P_1B_-Type heavy-metal ATPase, OsHMA9, is a metal efflux protein Plant Physiol 2007 145 831 842 10.1104/pp.107.102236 17827266 PMC2048805

[B15] Verret F Gravot A Auroy P Leonahardt N David P Nussaume L Vavasseur A Richaud P Overexpression of AtHMA4 enhances root-to-shoot translocation of zinc and cadmium and plant metal tolerance FEBS Lett 2004 576 306 312 10.1016/j.febslet.2004.09.023 15498553

[B16] Courbot M Willems G Motte P Arvidsson S Roosens N Saumitou-Laprade P Verbruggen N A major quantitative trait locus for cadmium tolerance in *Arabidopsis halleri* colocalizes with *HMA4*, a gene encoding a heavy metal ATPase Plant Physiol 2007 144 1052 1065 10.1104/pp.106.095133 17434989 PMC1914159

[B17] Kim YY Choi H Segami S Cho HT Martinoia E Maeshima M Lee Y AtHMA1 contributes to the detoxification of excess Zn(II) in Arabidopsis Plant J 2009 58 737 753 10.1111/j.1365-313X.2009.03818.x 19207208

[B18] Wong CKE Cobbett CS HMA P-type ATPases are the major mechanism for root-to-shoot Cd translocation in *Arabidopsis thaliana* New Phytol 2009 181 71 78 10.1111/j.1469-8137.2008.02638.x 19076718

[B19] Esterbauer H Cheeseman KH Determination of aldehydic lipidperoxidation products: malonaldehyde and 4-hydroxynonenal Methods Enzymol 1990 186 407 421 2233308 10.1016/0076-6879(90)86134-h

[B20] Lee SC Luan S ABA signal transduction at the crossroad of biotic and abiotic stress responses Plant Cell Environ 2012 35 53 60 10.1111/j.1365-3040.2011.02426.x 21923759

[B21] Epstein E The anomaly of silicon in plant biology Proc Natl Acad Sci USA 1994 91 11 17 10.1073/pnas.91.1.11 11607449 PMC42876

[B22] Yamaji N Ma JF Further characterization of a rice silicon efflux transporter, Lsi2 Soil Sci Plant Nutr 2011 57 259 264 10.1080/00380768.2011.565480

[B23] Epstein E Silicon Annu Rev Plant Physiol Plant Mol Biol 1999 50 641 664 10.1146/annurev.arplant.50.1.641 15012222

[B24] Savant NK Korndorfer GH Datnoff LE Snyder GH Silicon nutrition and sugarcane production: a review J Plant Nutr 1999 22 1853 1903 10.1080/01904169909365761

[B25] Ma JF Miyak Y Takahashi E Datonoff LF, Snyder GH, Korndorfer GH Silicon as a beneficial element for crop plants Silicon in agriculture 2001 Amsterdam: Elsevier Science Publishers 17 39

[B26] Liang YC Chen Q Liu Q Zhang WH Ding RX Exogenous silicon (Si) increases antioxidant enzyme activity and reduces lipid peroxidation in roots of salt-stressed barley (*Hordeum vulgare* L.) J Plant Physiol 2003 160 1157 1164 10.1078/0176-1617-01065 14610884

[B27] Liang YC Ding RX Influence of silicon on microdistribution of mineral ions in roots of salt-stressed barley as associated with salt tolerance in plants Sci China (Series C) 2002 45 298 308 10.1360/02yc9033 18759053

[B28] Hamayun M Sohn EY Khan SA Shinwari ZK Khan AL Lee IJ Silicon alleviates the adverse effects of salinity and drought stress on growth and endogenous plant growth hormones of soybean (*Glycine max* L.) Pak J Bot 2010 42 1713 1722

[B29] Chen W Yao X Cai K Chen J Silicon alleviates drought stress of rice plants by improving plant water status, photosynthesis and mineral nutrient absorption Biol Trace Elem Res 2011 142 67 76 10.1007/s12011-010-8742-x 20532668

[B30] Kim YH Khan AL Hamayun M Kang SM Beom YJ Lee IJ Influence of short-term silicon application on endogenous physiohormonal levels of *Oryza sativa* L. under wounding stress Biol Trace Elem Res 2011 144 1175 1185 10.1007/s12011-011-9047-4 21465280

[B31] Ma JF Yamaji N Mitani N Tamai K Konishi S Fujiwara T Katsuhara M Yano M An efflux transporter of silicon in rice Nature 2007 448 209 212 10.1038/nature05964 17625566

[B32] Hussain D Haydon MJ Wang Y Wong E Sherson SM Young J Camakaris J Harper JF Cobbett CS P-type ATPase heavy metal transporters with roles in essential zinc homeostasis in Arabidopsis Plant Cell 2004 16 1327 1339 10.1105/tpc.020487 15100400 PMC423219

[B33] Andrés-Colás N Sancenón V Rodríguez-Navarro S Mayo S Thiele DJ Ecker JR Puig S Peñarrubia L The Arabidopsis heavy metal P-type ATPase HMA5 interacts with metallochaperones and functions in copper detoxification of roots Plant J 2006 45 225 236 10.1111/j.1365-313X.2005.02601.x 16367966

[B34] Nocito FF Lancilli C Dendena B Lucchini G Sacchi GA Cadmium retention in rice roots is influenced by cadmium availability, chelation and translocation Plant Cell Environ 2011 34 994 1008 10.1111/j.1365-3040.2011.02299.x 21388416

[B35] Ma JF Yamaji N Mitani N Xu XY Su YH McGrath SP Zhao FJ Transporters of arsenite in rice and their role in arsenic accumulation in rice grain Proc Natl Acad Sci USA 2008 105 9931 9935 10.1073/pnas.0802361105 18626020 PMC2481375

[B36] Fujimaki S Suzui N Ishioka NS Kawachi N Ito S Chino M Nakamura S Tracing cadmium from culture to spikelet: noninvasive imaging and quantitative characterization of absorption, transport, and accumulation of cadmium in an intact rice plant Plant Physiol 2010 152 1796 1806 10.1104/pp.109.151035 20172965 PMC2850040

[B37] Shimo H Ishimaru Y An G Yamakawa T Nakanishi H Nishizawa NK *Low cadmium* (LCD), a novel gene related to cadmium tolerance and accumulation in rice J Exp Bot 2011 62 5727 5734 10.1093/jxb/err300 21908474 PMC3223062

[B38] Chien HF Kao CH Accumulation of ammonium in rice leaves in response to excess cadmium Plant Sci 2000 156 111 115 10.1016/S0168-9452(00)00234-X 10908811

[B39] McGrath SP Zhao FJ Lombi E Plant and rhizosphere processes involved in phytoremediation of metal contaminated soil Plant Soil 2001 232 207 214 10.1023/A:1010358708525

[B40] Sharma SS Dietz KJ The relationship between metal toxicity and cellular redox imbalance Trends Plant Sci 2009 14 43 50 10.1016/j.tplants.2008.10.007 19070530

[B41] Abdel-Ghany SE Müller-Moulé P Niyogi KK Pilon M Shikanai T Two P-type ATPases are required for copper delivery in *Arabidopsis thaliana* chloroplasts Plant Cell 2005 17 1233 1251 10.1105/tpc.104.030452 15772282 PMC1087999

[B42] Sudo E Itouga M Yoshida-Hatanaka K Ono Y Sakakibara H Gene expression and sensitivity in response to copper stress in rice leaves J Exp Bot 2008 59 3465 3474 10.1093/jxb/ern196 18676621 PMC2529235

[B43] Langer I Krpata D Fitz WJ Wenzel WW Schweiger PF Zinc accumulation potential and toxicity threshold determined for a metal-accumulating *Populus canescens* clone in a dose–response study Environ Pollut 2009 157 2871 2877 10.1016/j.envpol.2009.04.003 19446384

[B44] Liu C Li F Luo C Liu X Wang S Liu T Li X Foliar application of two silica sols reduced cadmium accumulation in rice grains J Hazardous Mat 2009 161 1466 1472 10.1016/j.jhazmat.2008.04.116 18555602

[B45] Baryla A Carrier P Franck F Coulomb C Sahut C Havaux M Leaf chlorosis in oilseed rape plants (*Brassica napus*) grown on cadmium-polluted soil: causes and consequences for photosynthesis and growth Planta 2001 212 696 709 10.1007/s004250000439 11346943

[B46] Chen HM Zheng CR Tu C Shen ZG Chemical methods and phytoremediation of soil contaminated with heavy metals Chemosphere 2000 41 229 234 10.1016/S0045-6535(99)00415-4 10819205

[B47] Shi XH Zhang CH Wang H Zhang FS Effect of Si on the distribution of Cd in rice seedlings Plant Soil 2005 272 53 60 10.1007/s11104-004-3920-2

[B48] da Cunha KPV do Nascimento CWA Silicon effects on metal tolerance and structural changes in maize (*Zea mays* L.) grown on a cadmium and zinc enriched soil Water Air Soil Poll 2009 197 323 330 10.1007/s11270-008-9814-9

[B49] Landberg L Greger M Differences in oxidative stress in heavy metal resistant and sensitive clones of *Salix viminalis* J Plant Physiol 2002 159 69 75 10.1078/0176-1617-00504

[B50] Morelli E Scarano G Copper-induced changes of non-protein thiols and antioxidant enzymes in the marine microalga *Phaeodactylum tricornutum* Plant Sci 2004 167 289 296 10.1016/j.plantsci.2004.04.001

[B51] Zhang H Xia Y Wang G Shen Z Excess copper induces accumulation of hydrogen peroxide and increases lipid peroxidation and total activity of copper-zinc superoxide dismutase in roots of *Elsholtzia haichowensis* Planta 2008 227 465 475 17909854 10.1007/s00425-007-0632-x

[B52] Davey MW Stals E Panis B Keulemans J Swennen RL High-throughput determination of malondialdehyde in plant tissues Anal Biochem 2005 347 201 207 10.1016/j.ab.2005.09.041 16289006

[B53] Weber H Chételat A Reymond P Farmer EE Selective and powerful stress gene expression in Arabidopsis in response to malondialdehyde Plant J 2004 37 877 889 10.1111/j.1365-313X.2003.02013.x 14996219

[B54] Upchurch RG Fatty acid unsaturation, mobilization, and regulation in the response of plants to stress Biotechnol Lett 2008 30 967 977 10.1007/s10529-008-9639-z 18227974

[B55] Molina A Bueno P Marín MC Rodríguez-Rosales MP Belver A Venema K Donaire JP Involvement of endogenous salicylic acid content, lipoxygenase and antioxidant enzyme activities in the response of tomato cell suspension cultures to NaCl New Phytol 2002 156 409 415 10.1046/j.1469-8137.2002.00527.x 33873571

[B56] He Y Liu Y Cao W Hua M Xu B Huang B Effects of salicylic acid on heat tolerance associated with antioxidant metabolism in Kentucky bluegrass Crop Sci 2005 45 988 995 10.2135/cropsci2003.0678

[B57] Shi Q Zhu Z Effects of exogenous salicylic acid on manganese toxicity, element contents and antioxidative system in cucumber Environ Exp Bot 2008 63 317 326 10.1016/j.envexpbot.2007.11.003

[B58] Metwally A Finkemeier I Georgi M Dietz KJ Salicylic acid alleviates the cadmium toxicity in barley seedlings Plant Physiol 2003 132 272 281 10.1104/pp.102.018457 12746532 PMC166972

[B59] Wang YS Wang J Yang ZM Wang QY Li B Li SQ Lu YP Wang SH Sun X Salicylic acid modulates aluminum-induced oxidative stress in roots of *Cassia tora* Acta Bot Sin 2004 46 819 828

[B60] Hey SJ Byrne E Halford NG The interface between metabolic and stress signaling Ann Bot 2010 105 197 203 10.1093/aob/mcp285 20007158 PMC2814758

[B61] Monni S Uhling C Hansen E Magel E Ecophysiological responses of *Empetrum nigrum* to heavy metal pollution Environ Poll 2001 112 121 129 10.1016/S0269-7491(00)00125-1 11234528

[B62] Gong HJ Randall DP Flowers TJ Silicon deposition in the root reduces sodium uptake in rice (*Oryza sativa* L.) seedlings by reducing bypass flow Plant Cell Environ 2006 29 1970 1979 10.1111/j.1365-3040.2006.01572.x 16930322

[B63] Yoshida S Ohnishi Y Kitagishi K Role of silicon in rice nutrition Soil Plant Food 1959 5 127 133 10.1080/00380768.1959.10430905

[B64] Brady AP Brown AG Huff H The polymerization of aqueous potassium silicate solutions J Colloid Sci 1953 8 2 252 276 10.1016/0095-8522(53)90043-9

[B65] Kaya C Tuna AL Sonmez O Ince F Higgs D Mitigation effects of silicon on maize plants grown at high zinc J Plant Nut 2009 32 1788 1798 10.1080/01904160903152624

[B66] Qi QG Rose PA Abrams GD Taylor DC Abrams SR Cutler AJ (?)-Abscisic acid etabolism, 3-ketoacylcoenzyme A synthase gene expression, and very-longchain monounsaturated fatty acid biosynthesis in *Brassica napus* embryos Plant Physiol 1998 117 979 987 10.1104/pp.117.3.979 9662540 PMC34952

[B67] Kamboj JS Browning G Blake PS Quinlan JD Baker DA Kamboj JS GC-MS SIM analysis of abscisic acid and indole-3-acetic acid in shoot bark of apple root stocks J Plant Growth Regul 1999 28 21 27

[B68] McCloud ES Baldwin IT Herbivory and caterpillar regurgitants amplify the wound-induced increases in jasmonic acid but not nicotine in *Nicotiana sylvestris* Planta 1997 203 430 435 10.1007/s004250050210

[B69] Enyedi AJ Yalpani N Silverman P Raskin I Localization, conjugation, and function of salicylic acid in tobacco during the hypersensitive reaction to tobacco mosaic virus Proc Natl Acad Sci USA 1992 89 2480 2484 10.1073/pnas.89.6.2480 1549613 PMC48682

[B70] Seskar M Shulaev V Raskin I Endogenous methyl salicylate in pathogen-inoculated tobacco plants Plant Physiol 1998 116 387 392 10.1104/pp.116.1.387

[B71] Ohkawa H Ohishi N Yagi K Assay for lipid peroxides in animal tissues by thiobabituric acid reaction Anal Biochem 1979 95 358 10.1016/0003-2697(79)90738-3 36810

