# Supplementary material for: Silicon mitigates heavy metal stress by regulating P-type heavy metal ATPases, Oryza sativa low silicon genes, and endogenous phytohormones
Source: BMC Plant Biol. 2014 Jan 9;14:13. doi: 10.1186/1471-2229-14-13 (PMC3893592; doi:10.1186/1471-2229-14-13)
Supplement: Additional file 1: Table S1 — HPLC conditions used for salicylic acid analysis. [file 1471-2229-14-13-S1.doc]

**Additional file 1: Table S1.** HPLC conditions used for salicylic acid analysis.

| **Gradient** | **5min (**%**)** | **2.5min (**%**)** | **4.5min (**%**)** | **5min (**%**)** | **3min (**%**)** |
| --- | --- | --- | --- | --- | --- |
| Solvent A | 30 | 40 | 60 | 30 | 30 |
| Solvent B | 70 | 60 | 40 | 70 | 70 |

Solvent A = 100% MeOH;

Solvent B = 100% water in 0.5% acetic acid
