# Supplementary material for: Silicon mitigates heavy metal stress by regulating P-type heavy metal ATPases, Oryza sativa low silicon genes, and endogenous phytohormones
Source: BMC Plant Biol. 2014 Jan 9;14:13. doi: 10.1186/1471-2229-14-13 (PMC3893592; doi:10.1186/1471-2229-14-13)
Supplement: Additional file 2: Table S2 — List of primers used for RT-PCR analysis. [file 1471-2229-14-13-S2.doc]

**Supply Table 2: List of primers used for RT-PCR analysis**

| **Name** | **Accession** | **Forward Primer (5´-3´)** | **Reverse Primer (5´-3´)** |
| --- | --- | --- | --- |
| *OsAct1* | NM001057621 | GTATCCATGAGACTACATACAACT | TACTCAGCCTTGGCAATCCACA |
| *OsHMA3* | AB559522.1 | CTCTGGTGATGCTTGTGAGC | TGATGCCCAGCGATCCAAGC |
| *OsHMA5* | NM001052568 | GGGGCTAATCATGGGGTACT | GAACTCCTGTCTTCGCGTTC |
| *OsLsi1* | NM001054643 | CGGTGGATGTGATCGGAACCA | CGTCGAACTTGTTGCTCGCCA |
| *OsLsi2* | HM055577 | ATCTGGGACTTCATGGCCC | ACGTTTGATGCGAGGTTGG |
|  |  |  |  |
